# A Longitudinal Case of Shared Delusional Infestation

**DOI:** 10.7759/cureus.34546

**Published:** 2023-02-02

**Authors:** Daniel Romine, Sarah Winston Bush, Joshua C Reynolds

**Affiliations:** 1 Emergency Medicine, Corewell Health West, Grand Rapids, USA; 2 Emergency Medicine, Michigan State University College of Human Medicine, Grand Rapids, USA

**Keywords:** emergency department, emergency medicine, shared psychotic disorder, delusory parasitosis, delusional infestation

## Abstract

Delusional infestation disorders are characterized by fixed but false beliefs about infection by a parasite, insect, or other living organism. Shared psychotic disorders are characterized by a single delusion instigated by a “primary” index patient and then induced in one or more “secondary” persons. We describe a case report of shared delusional infestation among an index patient and two family members that generated many healthcare encounters over the course of 12-15 months. This case report highlights the challenges in diagnosing and treating these conditions in the Emergency Department setting and their disproportionate utilization of healthcare resources. We discuss risk factors and characteristics of delusional infestations and shared psychotic disorders, as well as best practices to approach diagnosis, treatment, and disposition in the Emergency Department.

## Introduction

Shared psychotic disorders, originally termed “folie à deux” or Lasègue-Falret syndrome, are controversial and rare conditions characterized by a single delusion or hallucination instigated by a “primary” patient and induced in one or more “secondary” persons. These “secondary” persons typically have close associations with the primary patient. Literature on this condition is sparse. Charles Lasègue and Jules Falret first described shared psychotic disorders in the 1800s [1] and they have listed separately in the Diagnostic and Statistical Manual of Mental Disorders (DSM) 4th edition before being re-categorized under “other” schizophrenia/psychosis in the DSM-5 [2,3]. The prevalence and root causes are uncertain but known risk factors include being in a long-term relationship, female sex, advanced age, social isolation, stressful life events, impaired cognition, personality disorder, and schizophrenia [2].

Delusional infestations are classified in DSM-5 under somatic-type delusional disorders [3]. Delusional infestations are likely more common than shared delusions, with an estimated incidence of 1.9 cases per 100,000 person-years and estimated prevalence of 27.3 per 100,000 person-years [4,5]. Notably, shared delusions are observed in up to 5%-15% of patients with a delusional infestation [6].

Given the infrequent occurrence of these conditions, a single combined presentation is rare and described largely in case reports. There is no clear consensus on a treatment pathway for these patients. We describe the presentation and longitudinal clinical course of a patient with a combined shared psychotic disorder and delusional infestation.

## Case presentation

A 68-year-old female with underlying hypertension, hyperlipidemia, hypothyroidism, pulmonary embolism on apixaban, and no known substance use disorder initially presented to the emergency department (ED) in September of 2020 with a chief complaint “I have worms on my chin.” The patient described that an unspecified insect had bitten her chin approximately one year prior leaving a residual non-healing wound infested with “worms.” Recently, the patient’s granddaughter also reported seeing these worms emerging from her wound. The patient recounted three prior primary care physician (PCP) visits for her wound and used multiple topical treatments (silver sulfadiazine, antibiotic soap, and diclofenac cream) without improvement. Upon further review of medical records, her PCP had already prescribed courses of sulfamethoxazole-trimethoprim, cephalexin, and albendazole. In addition, s(he) had sent multiple wound specimens for pathology (all of which returned negative), attempted infectious disease (ID) referral (denied by the ID clinic based on negative pathology testing), attempted psychiatric referral (declined by the patient) and referred the patient to a wound clinic (patient canceled her appointments for fear that bandaging would trap the worms inside her wound). After bedside assessment and review of these previous healthcare encounters, the ED provider advised the patient she did not have a bacterial or parasitic infection, did not provide any medications, and encouraged PCP follow-up. The patient became agitated and left the ED.

The following day, the patient presented to a different ED stating “I am convinced I have worms in the wound on my face - no one will help me.” Her husband accompanied her for this visit, affirming that he had also seen the worms and tried to remove them whenever they “get aggressive and come out [sic],” but was unsuccessful because they “instantly deflate and are gone or go back in [sic].” At this ED visit, the provider ordered parasitic testing, provided additional topical silver sulfadiazine, and arranged a dermatology follow-up.

**Figure 1:**
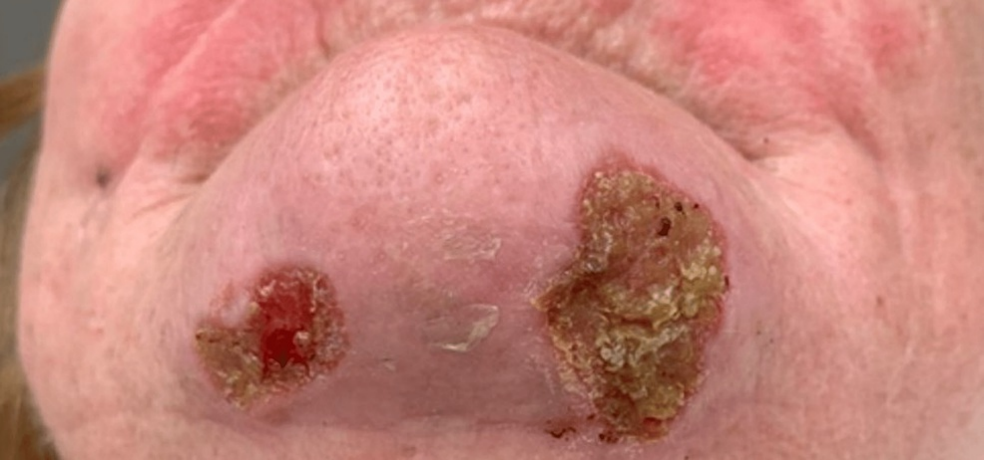
Ulcerative chin lesions

Several days later, the patient presented to a third ED accompanied by her husband. She expressed concern that the worms were now crawling through her body and were “eat[ing] my heart and brain.” She described unsuccessful attempts to remove the worms by scraping the wound with a hot butter knife and piercing the worms with needles and other sharp objects. The first photographic documentation of her wound was made at this time, revealing several excoriated and ulcerated lesions on her chin (Figure 1).

The patient reported visiting a dermatology clinic the previous day, but stated the dermatologist declined to biopsy her wound, and instead advised her that “she was delusional.” The patient was not receptive to this idea. She maintained that the worms were increasing in number and were now emerging from her eyes and her urine. The patient’s husband affirmed these allegations, stating he had also witnessed these occurrences. The ED provider offered psychiatric evaluation, which the patient refused, stating “It's not in my head. I don't need to see a counselor nor [sic] a psychiatrist." The ED provider contacted her PCP, who affirmed he was aware of the patient’s concern for worms and that he had again referred the patient to ID, psychiatry, and dermatology. Inpatient psychiatric assessment and treatment were considered at this ED visit, but upon further consideration and evaluation by behavioral health consultants, the patient was deemed to be at low risk for serious self-harm. The ED provider referred the patient back to a wound clinic and arranged another PCP appointment for a close follow-up. The ED provider also attempted to bandage the wound, but the patient refuse to cite that the worms would not be “able to breathe” and would “just make another hole.” The patient was prescribed sulfamethoxazole-trimethoprim mitigate the risk of wound infection and discharged home. She did visit the wound clinic the subsequent, but again refused wound care at that time.

In October 2020, the patient presented to yet another ED with concerns about “parasites under my skin.” At this visit, she was noted to have progressive scabbed, excoriated wounds on her neck as well as her chin. The ED provider contacted the PCP again and upon further discussion, scheduled yet another ID and dermatology appointment. The patient was prescribed topical mupirocin ointment and discharged home.

In late October 2020, the patient presented to her PCP office, at which point her PCP pursued inpatient psychiatric evaluation and treatment for a delusional disorder. She was transported to the nearest ED for medical screening, at which time, the ED provider noted progression of her wounds with new lesions on her arm as well. The patient reported continued attempts to scrape her wounds with a hot butter knife, and daily rinsing of the wounds with astringents, hydrogen peroxide, and other household cleaning agents to remove the worms. Upon direct questioning, she denied visual or auditory hallucinations and suicidal or harmful thoughts. She had no concern that any of her behaviors were abnormal. An institution-standard mental health laboratory panel for the purposes of medical screening (complete blood count, complete metabolic panel, thyroid stimulating hormone, acetaminophen and salicylate levels, and urine drug screen) prior to psychiatric hospitalization was unremarkable. The patient received a provisional diagnosis of delusional infestation and was transferred to an inpatient psychiatric facility.

Further notes are sparse, but the patient did have two additional ED visits in January 2021 for persistent wounds on her chin, at which point the provider documented she was “picking at them.” A review of PCP notes reveals multiple office visits throughout 2021 for wound-related issues. A review of a statewide prescription database reveals multiple subsequent courses of albendazole, amoxicillin/clavulanate, cefuroxime, cephalexin, clindamycin, doxycycline, mupirocin, silver sulfadiazine and sulfamethoxazole-trimethoprim from numerous providers throughout 2021. These items suggest continued complications of her wounds and/or an unresolved shared delusional infestation.

## Discussion

Delusional infestations pose a challenge to ED providers. These disorders are often a diagnosis of exclusion, lack clear acute diagnostic and treatment recommendations, and commonly generate recurrent visits with fragmented healthcare and disproportionate resource utilization [7]. When entertaining a diagnosis of delusional infestation, it is imperative to determine whether the patient has a “shakable” or “unshakable” belief in their infestation. In other words, ascertain how the patient would respond if all diagnostic testing were to conclude that an infestation was not present [8]. A patient receptive to the idea of a negative work-up is unlikely to have a true delusional disorder.

The differential diagnosis for organic causes of such symptoms includes traumatic brain injury, cerebrovascular disease, dementia, other cognitive impairments, thyroid dysfunction, nutritional deficiencies, diabetes, recreational drug intoxication, chronic substance abuse and/or withdrawal, drug reactions/allergies, human immunodeficiency virus, and actual parasitic infestations [9]. Initial diagnostic testing available in the ED includes complete blood count with differential (primarily to screen for eosinophilia); complete metabolic panel (including glucose, urea, and liver function); serum levels of thyroid stimulating hormone, B12, and folic acid; urine toxicology; syphilis screening; human immunodeficiency virus testing; and non-contrast computed tomography (CT) head [7]. Biopsy and pathology testing are typically deferred to the PCP or outpatient specialty clinic. If initial diagnostic testing is a negative and delusional disorder is suspected, the mainstay of treatment for delusional infestation is a strong and consistent relationship between the patient and a PCP. Within this patient-provider relationship, the provider works to build a therapeutic alliance by remaining non-judgmental, acknowledging but not necessarily agreeing with the delusions, and providing consistent reassurance of a negative workup with transparency of testing results (e.g., serum laboratory testing, pathology, imaging, etc.) [7]. Any patient diagnosed with a delusional disorder should be carefully assessed for comorbid pre-existing or superimposed psychiatric conditions.

Some patients with delusional infestations prove resistant to these initial steps. Subsequent treatments for delusional infestations include pharmacotherapy with anti-depressants or anti-psychotics, counseling, and cognitive behavioral therapy. In this case report, the patient had a consistent follow-up with her PCP, yet resisted attempts at referral for outpatient psychiatric resources and had multiple visits to different EDs, dermatology, infectious disease, and wound clinics, before finally being admitted to an inpatient psychiatric facility. Furthermore, her office notes and prescription records suggest that despite her inpatient psychiatric hospitalization, her delusion persisted into the next calendar year. We note that we find no record of the patient receiving any antipsychotic medications. Although they are rarely prescribed in the ED, antipsychotics are associated with improved clinical outcomes in cases of delusional infestation with up to 60%-100% effectiveness [10,11].

This case was further complicated by evidence of shared delusion between the patient (primary), her husband, and her granddaughter. Because the patient’s husband and granddaughter were never registered as patients for an ED healthcare encounter, we are not privy to their medical and psychiatric histories. Treatment for a shared delusional infestation is similar to treatment of single delusional infestations in that it is predicated on a strong and regular relationship with a PCP characterized by the features previously noted. Antipsychotic medications are also effective in shared delusions. In persistent cases, the primary patient is separated from the secondary person(s) [12]. It is unclear from the available documentation if the shared psychotic disorder aspect of this case was ever addressed by a provider. It is likely that the shared delusion component of this case contributed to this patient’s resistance to treatments. 

Morgellons’s disease (MD) is a phenomenon characterized by a belief that parasites or fibers are emerging from the skin and associated sensations of crawling or stinging on or under the skin. Patients with MD commonly produce physical evidence of the infestation in the form of fibers or particles. Histopathological testing of submitted material variably identifies contents of cellulose, keratin, collagen, or assorted fabric fibers without evidence of an infectious component [13]. Some theorize that MD is the manifestation of an inflammatory overproduction of keratin and collagen with an organic etiology [14], whereas others note that MD is merely a rapport-enhancing term for delusional infestations [13,15]. In this case report, the patient displayed some features of MD, but also alleged systemic infestation with spread to other organs beyond her skin and did not produce physical specimens of the alleged worms.

## Conclusions

Delusional infestations pose a challenge for ED providers to strike a balance of duly evaluating for organic causes of the symptoms while tactfully encouraging the patient to participate in a diagnostic evaluation that does not propagate their delusions. Even if the delusional infestation is strongly suspected, a patient rarely poses a material risk of self-harm that necessitates emergent psychiatric hospitalization. The *de facto* disposition of discharge with outpatient follow-up often generates subsequent ED or specialist visits with disproportionate utilization of healthcare resources. ED providers best prepare the patient for outpatient follow-up by laying the groundwork for therapeutic alliance: remaining non-judgmental, acknowledging the delusions without agreeing with them, communicating in a clear and direct fashion, and providing reassurance with transparency in testing results. ED providers should also be alert to the presence of a shared delusion among secondary persons and employ the same tactics with them.
